# Immunophenotypic expression of UCP1 in hibernoma and other adipose/non adipose soft tissue tumours

**DOI:** 10.1186/s13569-019-0118-1

**Published:** 2019-05-13

**Authors:** Jessica Malzahn, Afroditi Kastrenopoulou, Ioanna Papadimitriou-Olivgeri, Dionysios J. Papachristou, Jennifer M. Brown, Udo Oppermann, Nick A. Athanasou

**Affiliations:** 1 Botnar Research Centre, Nuffield Department of Orthopaedics, Rheumatology and Musculoskeletal and Sciences, University of Oxford, Nuffield Orthopaedic Centre, Oxford, OX7 HE UK; 2 Laboratory of Bone and Soft Tissue Studies, Department of Anatomy-Histology-Embryology, University Patras Medical School, Patras, Greece

**Keywords:** UCP1, Brown fat, Hibernoma, Liposarcoma

## Abstract

**Background:**

Uncoupling protein 1 (UCP1) is a mitochondral protein transporter that uncouples electron transport from ATP production. UCP1 is highly expressed in brown adipose tissue (BAT), including hibernomas, but its expression in other adipose tumours is uncertain. UCP1 has also been found in other tissues (e.g. smooth muscle) but whether it is expressed in non-adipose benign and malignant soft tissue tumours is unknown.

**Methods:**

Immunohistochemical staining of normal (axillary) BAT and subcutaneous/abdominal white adipose tissue (WAT) as well as a wide range of benign and malignant primary soft tissue tumours (n = 171) was performed using a rabbit polyclonal antibody to UCP1. BAT and hibernomas were also stained by immunohistochemistry with monoclonal and polyclonal antibodies to adipose/non-adipose tumour markers in order to characterise the immunophenotype of BAT cells.

**Results:**

UCP1 was strongly expressed in the cytoplasm of brown fat cells in BAT and hibernomas, both of which also expressed aP2, S100, CD31, vimentin and calponin. UCP1 was not expressed in WAT or other adipose tumours with the exception a few tumour cells in pleomorphic liposarcoma. UCP1 was variably expressed by tumour cells in a few non-adipose sarcomas including leiomyosarcoma, rhabdomyosarcoma, alveolar soft part sarcoma, synovial sarcoma and clear cell sarcoma.

**Conclusions:**

UCP1 is strongly expressed in BAT but not WAT and is found in all hibernomas and a few pleomorphic liposarcomas but not in other adipose tumours. UCP1 expression in a few non-adipose soft tissue sarcomas may possibly reflect origin of tumour cells from a common mesenchymal stem cell precursor and/or developmental pathway.

## Background

Benign and malignant tumours of adipose differentiation represent a large category of soft tissue tumours, many of which are frequently encountered in diagnostic pathology [1]. These tumours contain neoplastic cells that show morphological evidence of adipose differentiation, notably the presence of cytoplasmic fat vacuoles and expression of adipocyte lineage markers such as aP2 [2–5]. White adipose tissue (WAT) cells in a lipoma have a large single lipid vacuole, whereas brown adipose tissue (BAT) cells in a hibernoma contain multiple small lipid vacuoles. In specific subtypes of benign and malignant adipose tumours there may be lipoblasts and/or atypical adipose cells with large or small, single or multiple, fat vacuoles as well as cells with pleomorphic or spindle-shaped nuclei [1].

Uncoupling proteins (UCPs) comprise a class of mitochondrial proteins that act to dissipate the proton gradient across the mitochondrial membrane; protons are transported from the inner membrane space back to the matrix, the energy lost in dissipating the proton gradient being generated as heat [6–8]. This process bypasses ATP synthase, an adjacent mitochondrial membrane proton channel that establishes the electrochemical gradient for ATP synthesis via oxidative phosphorylation. UCP1 (thermogenin), was the first UCP to be identified [6, 7]. BAT cells, which are specialised thermogenic cells, contain abundant UCP1. In contrast, WAT cells, which store lipids as energy are UCP1 negative. There is evidence that WAT can be converted into BAT (browning of WAT), and that within WAT there are “beige” adipocytes which express UCP1 [9, 10]. UCP1 expression has also been reported in non-adipose cells such as those of smooth and striated muscle and in cultures of adipose cells expressing muscle markers [11–13].

UCP1 is highly expressed in hibernoma, a tumour containing abundant BAT cells [14–17]. There are a few reports of UCP1 expression by tumour cells in liposarcoma [18, 19], but immunophenotypic expression of UCP1 in malignant adipose tumours has not been characterised. In this study, we have sought to determine whether UCP1 is expressed in specific benign and malignant adipose tumour subtypes. In addition, as UCP1 has been reported in other cell types, we have examined whether UCP1 is expressed in a wide range of non-adipose benign and malignant soft tissue tumours. We have also determined whether tumour markers other than UCP1 can be used to distinguish hibernoma from other adipose/non-adipose soft tissue tumours.

## Methods

### Human tissue samples

Samples of axillary adipose tissue that contained brown fat cells were employed as BAT-positive controls and subcutaneous (thigh) and mesenteric adipose tissue as WAT -positive controls. Bone marrow adipose tissue from cancellous bone of a lumbar vertebra and a femur were also examined. Other non-neoplastic tissues stained with UCP1 included sections of normal skin, appendix, bone, lymph node and tonsil. Paraffin-embedded tissue sections from formalin-fixed samples of WAT and BAT controls and 171 soft tissue tumours, retrieved from the files of the Nuffield Orthopaedic Centre, Oxford were analysed in this study. Negative controls for UCP1 staining consisted of BAT-positive control tissue sections stained without the primary antibody. The number and nature of the tumours examined in this study are shown in Table 1. This study was approved by the Oxford Clinical Research Ethics Committee (C01.071).

**Table 1:** Immunohistochemical staining for UCP1 in soft tissue tumours

Soft tissue tumours	Number of cases	UCP1 + cases
Adipose tumours		
Lipoma	16	0
Intramuscular lipoma	2	0
Hibernoma	17	17
Lipoblastoma	6	0
Spindle cell/pleomorphic lipoma	6	0
Angiolipoma	2	0
Chondroid lipoma	2	0
Atypical lipomatous tumour/well differentiated liposarcoma	20	0
Myxoid liposarcoma	6	0
Pleomorphic liposarcoma	6	2
Dedifferentiated liposarcoma	6	0
Other mesenchymal tumours		
Fibromatosis	2	0
Nodular facsciitis	2	0
Myxoma	2	0
Angioleiomyoma	2	0
Tenosynovial giant cell tumour	2	0
Leiomyoma^a^	6	0
Benign schwannoma	3	0
Ganglioneuroma	1	0
Neurofibroma	3	0
Malignant schwannoma	5	0
Fibromyxoid sarcoma	2	0
Myxofibrosarcoma	4	0
Ossifying fibromyxoid tumour	1	0
Solitary fibrous tumour	2	0
Haemangioma	2	0
Angiosarcoma	2	0
Alveolar soft part sarcoma	2	1
Clear cell sarcoma	2	0
Synovial sarcoma (monophasic)	4	1
Ewing sarcoma	5	0
Dermatofibrosarcoma protuberans	4	0
Leiomyosarcoma	6	2
Pleomorphic rhabdomyosarcoma	4	1
Alveolar rhabdomyosarcoma	4	1
Embryonal rhabdomyosarcoma	3	1
Granular cell tumour	2	0
Epithelioid sarcoma	3	0
Osteosarcoma	2	0

^a^Includes 3 uterine and 3 soft tissue lesions

### Immunohistochemistry

Four micrometre sections were cut and immunohistochemical staining was carried out using an Envision FLEX Minikit and the Dako Autostainer (Dako Agilent UK) as previously described [14, 19, 20]. The anti-UCP1 polyclonal rabbit antibody (diluted 1:250, 1: 500 (recommended dilution), 1:1000, 1:2000, 1:100000, 1:20000) employed in this study (ab10983) was obtained from Abcam, UK. Immunohistochemical staining with a range of other monoclonal and polyclonal antibodies to known tumour-related antigens (Table 2) was similarly carried out on sections of hibernoma and BAT controls.

**Table 2:** Details of monoclonal and polyclonal antibodies employed for immunohistochemical staining of BAT and hibernoma

Antibody (source)	Dilution	Antigen	Immunohistochemical staining
1A4 (Agilent, UK)	1/50	Smooth muscle actin (SMA)	−
HHF 35 (Agilent, UK)	1/50	Muscle specific actin	−
DE-R11 (Agilent, UK)	1/100	Desmin	−
h-CD (Agilent, UK)	1/200	Caldesmon	−
CALP (Agilent, UK)	1/50	Calponin	+
JC70 (Agilent, UK)	1/20	CD31	+
QBEnd 10 (Agilent, UK)	1/50	CD34	−
PD7/26 (Agilent, UK)	1/50	CD45	−
KP1 (Agilent, UK)	1/1000	CD68	−
Anti S100^a^ (Agilent, UK)	1/50	S100	++
Anti-FABP4 (Sigma, UK)	1/2500	aP2/FABP4	++
Anti-MIC2 (127E) (Leica, UK)	1/50	CD99	−
AE1/3 (Leica, UK)	1/50	Pan-cytokeratin	−
E29 (Aligent, UK)	1/500	Epithelial membrane antigen	−
A103 (Aligent, UK)	1/50	Melan A	−
D2-40 (Aligent, UK)	1/100	Podoplanin	−
Ab10983 (Abicam, UK)	1/250–1/20,000^a^	UCP1	++

^a^See “Results” for details

−: No staining

+: Weak staining

++: Strong staining

## Results

### UCP1 expression in normal BAT, WAT and other control tissues

**Fig. 1:**
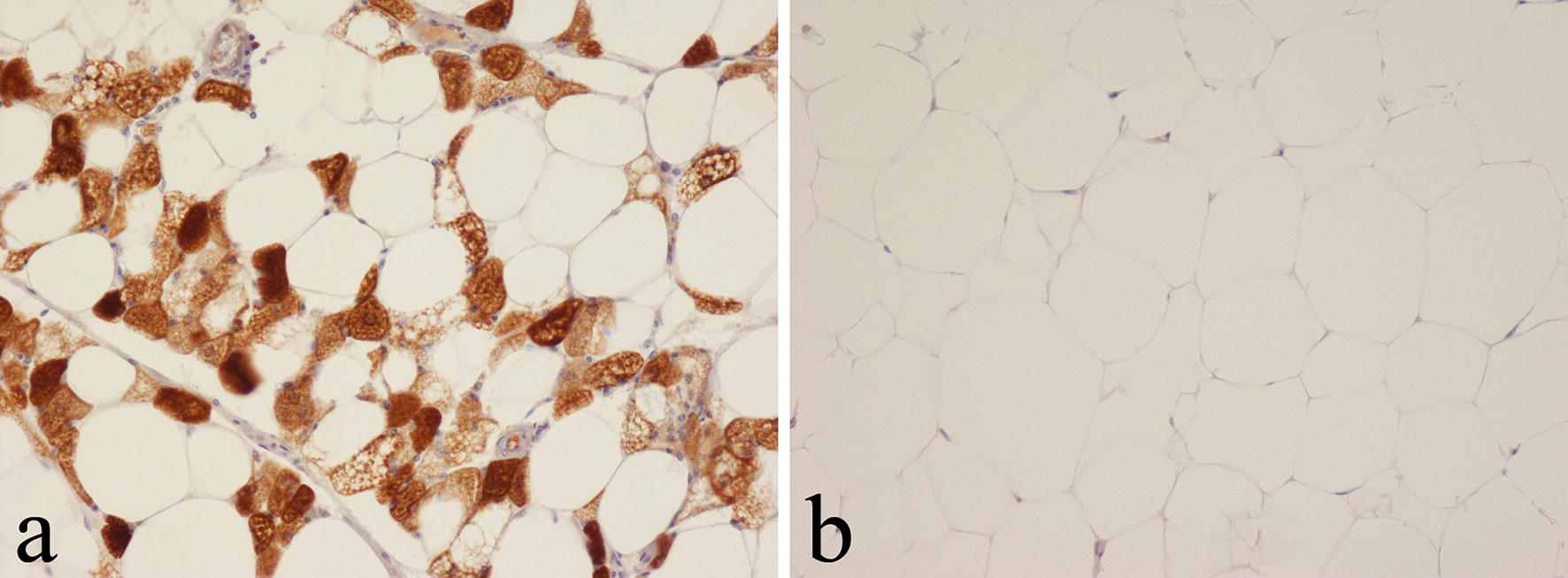
Immunohistochemical staining with the polyclonal anti-UCP1 antibody in: **a** axillary and **b** subcutaneous adipose tissue, showing positive staining of BAT but not WAT cells

In axillary adipose tissue there was strong staining of the multiple small fat vacuoles in the cytoplasm of brown fat cells with the anti-UCP1 polyclonal antibody; this was noted even when a low concentration (1:1000–1:20000) of the antibody was employed (Fig. 1a). In contrast, fat cells in subcutaneous, intra-abdominal and bone marrow WAT were negative even when the anti-UCP1 antibody was used at a high concentration (1:250) (Fig. 1b). Nuclei of both WAT and BAT cells were unstained. Connective tissue cells in normal WAT and BAT and other cells in normal control tissues (tonsil, appendix, bone, lymph node, skin) showed no staining with the anti-UCP1 antibody.

### UCP1 expression in benign and malignant adipose tumours

**Fig. 2:**
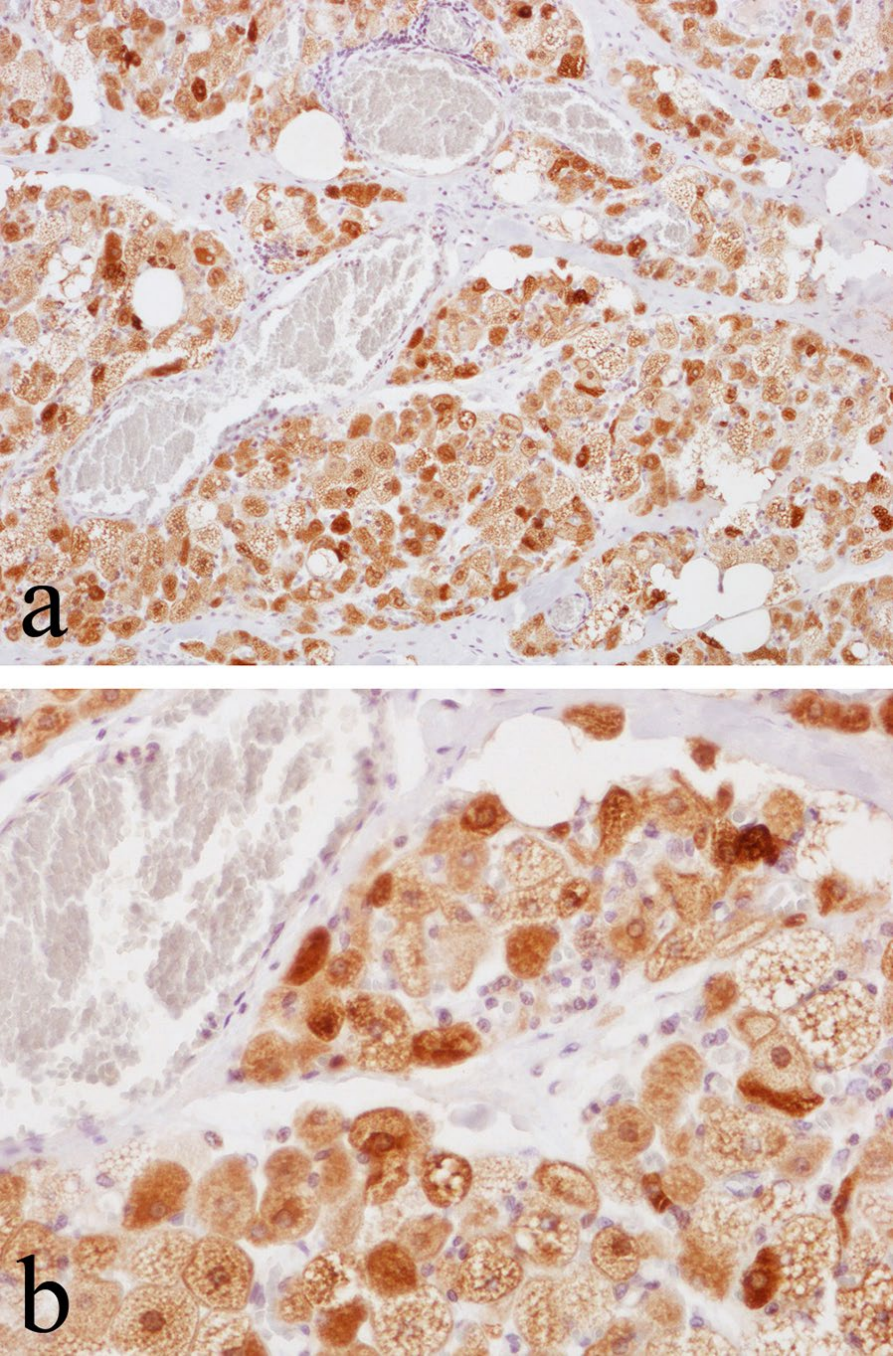
Immunohistochemical staining with the polyclonal anti-UCP1 antibody (1:20000 dilution), showing strong staining of BAT cells in hibernoma at **a** low power and **b** high-power

Brown fat cells in hibernoma stained strongly with the anti-UCP1 antibody (i.e. up to 1:20000 dilution) in a manner similar to that noted for axillary BAT (Fig. 2). In additon to UCP1, brown fat cells in both BAT and hibernomas strongly expressed aP2, vimentin, and S100 as well as more weakly expressing CD31 and calponin (Table 2). There was no expression of CD34, desmin, smooth muscle/muscle actin, CD99, podoplanin, EMA, cytokeratin, CD45, Melan A or caldesmon.

**Fig. 3:**
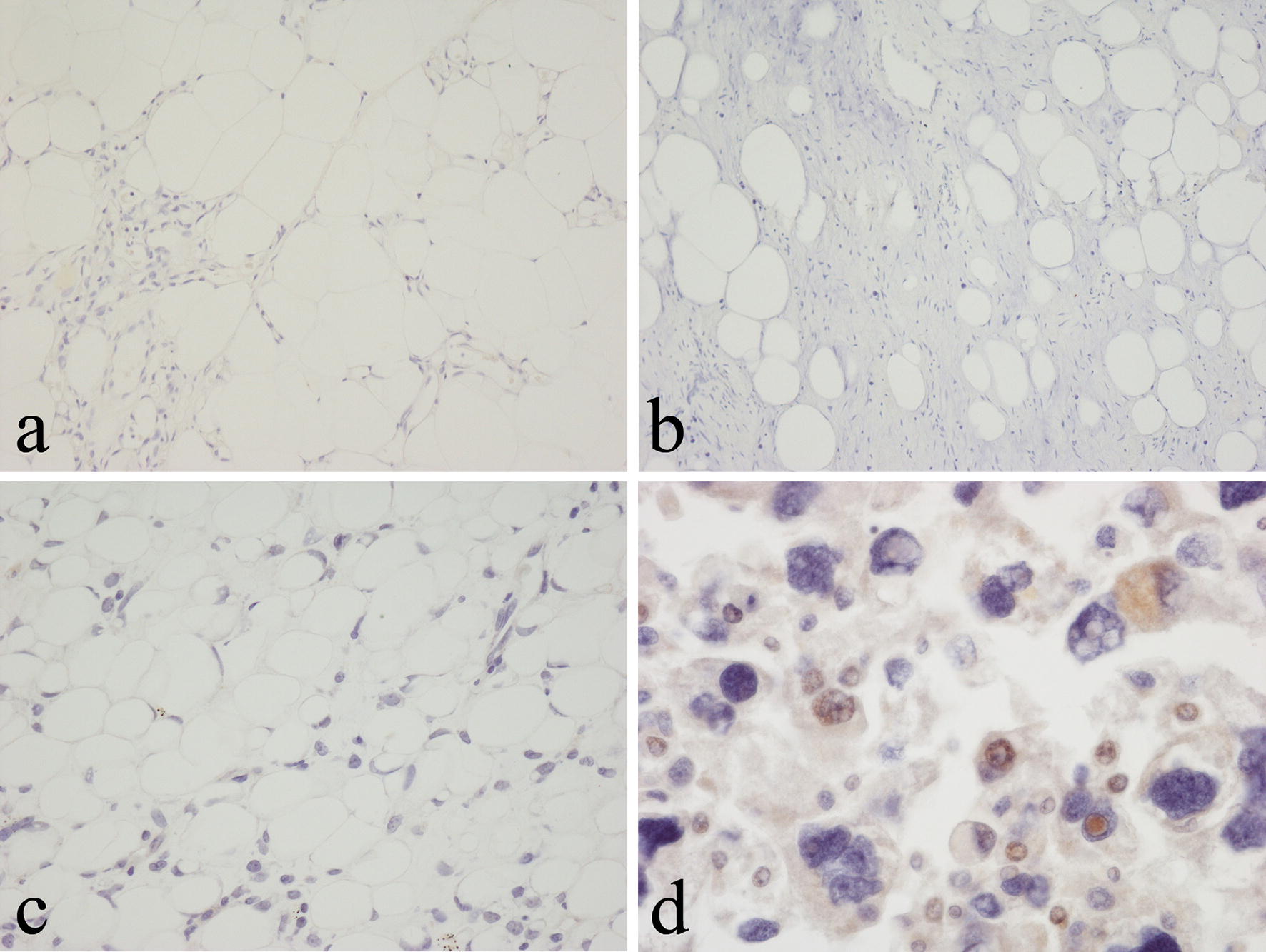
Immunohistochemical staining with the polyclonal anti-UCP1 antibody showing absence of staining in **a** angiolipoma, **b** spindle cell lipoma and **c** well-differentiated liposarcoma. **d** There is positive staining in pleomorphic liposarcoma

UCP1 expression was not seen in benign lipomas including those containing non-adipose connective tissue elements such as fibrolipoma and angiolipoma (Fig. 3a). Myxoid and lipomatous components of chondroid lipoma and lipomatous and spindle/pleomorphic cell components of spindle cell/pleomorphic lipoma were UCP1 negative (Fig. 3b). Both lipoblasts and WAT cells in lipoblastoma were UCP1-negative. No expression of UCP1 was noted in sections of atypical lipomatous tumour (ALT)/well-differentiated liposarcoma in extra-abdominal soft tissues or the retroperitoneum (Fig. 3c). Vacuolated small fat cells and lipoblast-like cells in myxoid liposarcoma did not stain for UCP1. Both the well-differentiated liposarcoma and malignant spindle cell sarcoma components of dedifferentiated liposarcoma were negative for UCP1. Most high-grade pleomorphic liposarcomas did not express UCP1 but in a minority of pleomorphic liposarcomas, scattered small and large vacuolated tumour cells expressed UCP1 (Fig. 3d).

### UCP1 expression in non-adipose soft tissue tumours

**Fig. 4:**
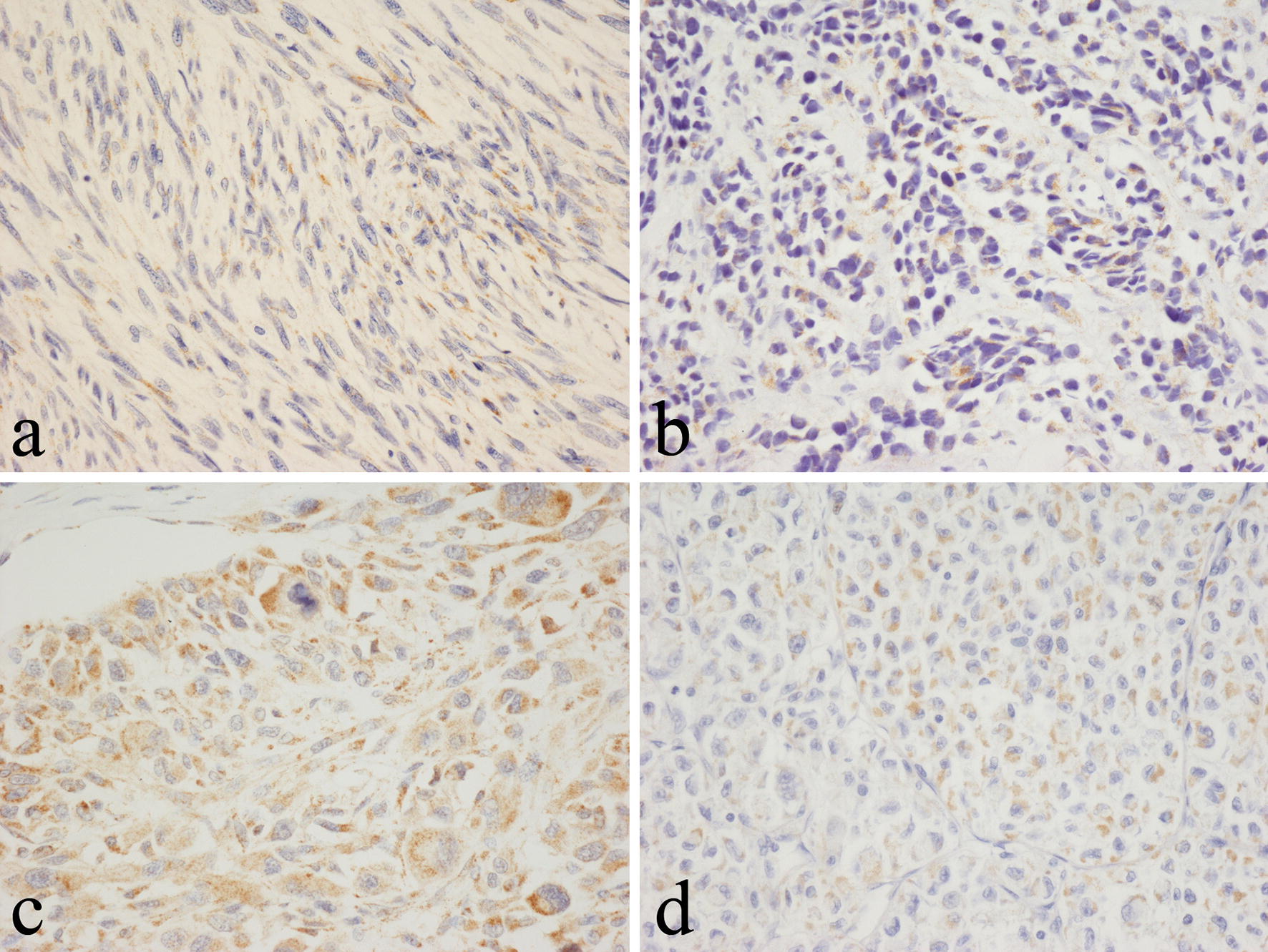
Immunohistochemical staining with the polyclonal anti-UCP1 antibody showing positive staining in **a** leiomyosarcoma, **b** alveolar rhabdomyosarcoma, **c** pleomorphic rhabdomyosarcoma and **d** alveolar soft part sarcoma

UCP1 expression was not seen in any of the benign (non-adipose) soft tissue tumours examined, including those of smooth muscle, nerve sheath and vascular differentiation. Both uterine and soft tissue leiomyomas were UCP1-negative. Benign fibrohistiocytic tumours and fibroblastic/myofibroblastic lesions such as myofibroma, nodular fascitiis and fibromatosis were also UCP1 negative. Most malignant (non-adipose) mesenchymal soft tissue neoplasms (Table 1) did not stain for UCP1, but it was noted that tumour cells in a few leiomyosarcomas and alveolar and pleomorphic rhabdomyosarcomas stained strongly for UCP1 when the polyclonal antibody was diluted 1:500 or less (Fig. 4a–c). Weak staining for UCP1 at this dilution was also noted in some tumour cells of a few other soft tissue sarcomas including alveolar soft part sarcoma (Fig. 4d), biphasic synovial sarcoma and undifferentiated pleomorphic sarcoma.

## Discussion

Tumours of adipose differentiation represent a large category of mesenchymal soft tissue tumours with lipoma and liposarcoma being respectively the most common benign and malignant soft tissue tumours diagnosed in adults. In this study we have shown that UCP1 is strongly expressed in BAT but not WAT and that, with the exception of all hibernomas and a minority of pleomorphic liposarcomas, UCP1 is not expressed in benign and maligant adipose tumours. UCP1 was not expressed in non-adipose tissues and was not seen in benign, non-adipose soft tissue tumours, but was found in some non-adipose soft tissue sarcomas, notably rhabdomyosarcoma and leiomyosarcoma.

It has been shown that normal WAT cells can differentiate into BAT cells to form beige/brite cells [9, 10]. Schulz et al. [12] noted that human pre-adipocytes isolated from subcutaneous WAT have a greater inducible capacity to become BAT cells compared with those isolated from mesenteric or omental WAT. We found no immunophenotype evidence of UCP1 expression in normal subcutaneous or intra-abdominal WAT even when a high concentration of the anti-UCP1 polyclonal antibody was used. We also noted no evidence of UCP1 in bone marrow WAT. Bone marrow adipocytes store significant quantities of fat and are thought to play a role in systemic energy metabolism with functional BAT-like cells having been described in bone marrow fat [21]. Our findings indicate that BAT cells are not found in the marrow of bones of the axial or appendicular skelton; this finding is consistent with the observation that hibernomas have only rarely been reported in bone [22, 23].

In contrast to BAT cells in hibernoma, mature WAT cells in simple benign lipoma and in the various lipoma subtypes were UCP1-negative. UCP1-mRNA expression in lipomas of multiple symmetric lipomatosis, bearing pathogenic mitochondrial mutations has been reported, but UCP1 expression in simple lipomas or lipoma subtypes has not been identified [24]. Lipoblasts and WAT cells in lipoblastoma as well as in atypical lipoamtous tumour/well-differentiated liposarcoma were also negative for UCP1. Hibernoma-like cells have been described in well-differentiated liposarcoma and myxoid liposarcoma [25, 26], but we did not identify UCP1 expression in these lesions. Both the well-differentiated liposarcoma and spindle cell sarcoma components of dedifferentiated liposarcoma were UCP1 negative. Tumour cells in most high-grade pleomorphic liposarcomas were UCP1 negative, but it was noted that vacuolated tumour cells in a few pleomorphic liposarcomas stained for UCP1. WAT is the predominant form of adipose tissue present in humans after infancy so it is perhaps not surprising that most adipose tumours are composed of cells containing (UCP1-negative) WAT cells. Absence of UCP1 staining in most adipose tumours would also indicate that ‘browning’ of WAT to form beige/brite cells is not seen to any great extent. Our results contrast with the findings of La Doucer et al. [18], who noted not only that canine well-differentiated liposarcomas express UCP1 but also that a number of such tumours express smooth muscle actin and desmin with one case expressing myogenin; in our experience, these antigens are rarely expressed in well-differentiated or other subtypes of liposarcoma.

UCPs are mitochondrial membrane proteins that enable a rapid backflow of protons through the mitochondrial inner membrane, bypassing ATP synthase. This mitochondrial uncoupling is mediated by UCP1 and its homologues [6–8, 27]; the latter include UCP2, which is ubiquitously expressed, UCP3, which is known to be expressed in skeletal muscle, and UCP4 and UCP5, both of which are expressed in the central nervous system. Polyclonal antibodies can cross-react with structurally-related molecules [28] so it is possible that staining with the anti-UCP1 antibody of malignant tumour cells in some non-adipose sarcomas could represent a cross-reaction with a UCP1 homologue, such as UCP2 and/or UCP3 in rhabdomyosarcomas. Such cross reactions were not, however, seen in normal control (non-adipose) tissues or in benign (non-adipose) tumours. Another possible reason for some rhabdomyosarcomas expressing UCP1 is that brown fat and skeletal muscle have been shown to share a common developmental pathway. Brown fat progenitors have been identified in skeletal muscle [12, 29–31], and neoplastic cells in canine orbital hibernoma have been reported to express myogenin and myo-D1 [32]. Similarly, with regard to the immunophenotypic expression of UCP1 noted in some leiomyosarcomas, there is evidence from ribosomal profiling that a subset of beige fat cells are of smooth muscle-like origin; it has also previously been reported that UCP1 mRNA is expressed in uterine smooth muscle cells [11, 13]. These findings, taken with our results, would indicate that expression of UCP1 is not entirely specific for the differentiation of brown fat. It has been shown that ectopic expression of UCP1 can be induced in pluripotent stem cells [33]. It is possible that expression of UCP1 in rhabdomyosarcoma, leiomyosarcoma and in a few other sarcomas may reflect origin of tumour cells from a mesenchymal stem cell precursor exhibiting an uncoupled mitochondrial phenotype.

As in normal BAT, strong diffuse staining for UCP1 was seen in all hibernomas even when a low concentration (1:20000) of the anti-UCP1 antibody was employed. BAT cells in hibernoma have been shown to express vimentin, S100, aP2 and CD31 [4, 5, 26, 34]. In this study we report that calponin is also expressed in hibernoma (and in BAT). This finding is consistent with expression of smooth muscle antigens in BAT cells [11, 13] and in some lipoma subtypes [26, 35]. Hibernoma, however, is distinguished from lesions such as spindle cell/pleomorphic lipoma and haemosiderin fibrohistiocytic lipomatous tumour by the absence of CD34 expression and staining for UCP1 (at high dilution of the antibody). It is sometimes necessary, particularly in a fine needle biopsy where there is limited histological material for examination, to distinguish BAT cells in a hibernoma from ‘BAT-like’ neoplastic/non-neoplastic cells with vacuolated/foamy cytoplasm in other lesions; our findings showing an absence of expression of specific epithelial, melanoma, leucocyte, macrophage and other markers by BAT cells (taken with expression of aP2/FABP4 and UCP1), are of diagnostic utility in this regard.

## Conclusions

UCP1 is strongly expressed in BAT but not WAT, and is found in all hibernomas. Absence of UCP1 expression in other adipose tumours (with the exception of a few pleomorphic liposarcomas) suggests that these tumours are derived from WAT. UCP1 expression in a few non-adipose soft tissue sarcomas may possibly reflect origin of tumour cells from a common mesenchymal stem cell precursor and/or developmental pathway.

## Data Availability

Not applicable.

## Abbreviations

ALT: atypical lipomatous tumour
aP2: adipocyte protein 2
ATP: adenosine triphosphate
BAT: brown adipose tissue
EMA: epithelial membrane antigen
WAT: white adipose tissue
UCP1: uncoupling protein1
